# Incorrect measurements and misleading conclusions in the article “Comparison of the efficacy of tooth alignment among lingual and labial brackets: an in vitro study”

**DOI:** 10.1186/s13005-020-00221-7

**Published:** 2020-04-22

**Authors:** Dirk Wiechmann, Hans-Peter Bantleon, Birte Melsen, Björn Zachrisson, Urban Hägg, Pierre Canal, Robert Garcia, Stephane Barthélemi, Laure Frapier, Dan Grauer, Christian Sander, Peter Diedrich, Collin Jacobs, Heiner Wehrbein, Ariane Hohoff, Hans-Joachim Helms, Rainer Schwestka-Polly

**Affiliations:** 1 Senior Lecturer, Department of Orthodontics, Hannover Medical School, Carl-Neuberg-Str. 1, Hannover, D-30625 Germany; 2 Professor Emeritus, Medical University of Vienna, Spitalgasse 23, Vienna, 1090 Austria; 3 Adjunct Professor at University of Western Australia, 35 Stirling Highway, Perth WA, 6009 Australia; 4 Visiting Professor, New York University, 345 E. 24th Street, New York, NY, 10010 USA; 5 Professor Emeritus, Department of Orthodontics, University of Oslo, Boks 1072 Blindern, Oslo, NO-0316 Norway; 6 Emeritus and Honorary Professor, University of Hong Kong, The Prince Philip Dental Hospital, 34 Hospital Rd, Hong Kong, China; 7 Professeur des Universités Émérite à la Faculté d’Odontologie de Montpellier, 545 Avenue Pr JL Viala, Montpellier, 34193 France; 8 Professeur Émérite, UFR d’Odontologie, Université de Paris 5, Rue Garancière, Paris, 75006 France; 9 Professor, Department of Orthodontics & Dentofacial Orthopedics, University of Montpellier, 545 Avenue Pr JL Viala, Montpellier, 34193 France; 10 Head of Department, Department of Orthodontics & Dentofacial Orthopedics, University of Montpellier, 545 Avenue Pr JL Viala, Montpellier, 34193 France; 11 Adjunct Professor, Department of Orthodontics, University of North Carolina, Campus Box 7450, Chapel Hill, NC, 27599-7450 USA; 12 Member of the University of Ulm, Eversbuschstr. 107, Munich, 80999 Germany; 13 Director Emeritus, Clinic of Orthodontics, Medical Faculty RWTH Aachen, Pauwelsstrasse 30, Aachen, 50074 Germany; 14 Head of Department, Department of Orthodontics, Section of Preventive and Pediatric Dentistry, University Dental School of Jena, An der Alten Post 4, Jena, D-07743 Germany; 15 Head of Department, Department of Orthodontics, University Medical Center, Johannes Gutenberg University Mainz, Augustusplatz 2, Mainz, D-55131 Germany; 16 Head of Department, Department of Orthodontics, University of Münster, Albert-Schweitzer-Campus 1, Münster, D-48149 Germany; 17 Dr. rer. nat., Department of Medical Statistics, University Medical Center Göttingen, Humboldtallee 32, Göttingen, D-37073 Germany; 18 Head of Department, Department of Orthodontics, Hannover Medical School, Carl-Neuberg-Str. 1, Hannover, D-30625 Germany

**Keywords:** Lingual orthodontics, Lingual appliances, Lingual brackets, Orthodontic brackets, Orthodontic wires, Tooth movement techniques

## Abstract

**Background/objective:**

To reproduce the methods and results of the study by Alobeid et al. (2018) in which the efficacy of tooth alignment using conventional labial and lingual orthodontic bracket systems was assessed.

**Materials/methods:**

We used the identical experimental protocol and tested (i) regular twin bracket (GAC-Twin [Dentsply]) and lingual twin bracket systems (Incognito [3M]), (ii) together with NiTi 0.014” wires (RMO), and (iii) a simulated malocclusion with a displaced maxillary central incisor in the x-axis (2 mm gingivally) and in the z-axis (2 mm labially).

**Results:**

The method described by Alobeid et al. (2018) is not reproducible, and cannot be used to assess the efficacy of tooth alignment in labial or lingual orthodontic treatment. Major flaws concern the anteroposterior return of the Thermaloy-NiTi wire ligated with stainless steel ligatures. The reproduced experimental setting showed that a deflected Thermaloy-NiTi wire DOES NOT move back at all to its initial stage (= 0 per cent correction) because of friction and binding (see supplemented video), neither with the tested labial nor with the lingual brackets. Furthermore, an overcorrection of up to 138 per cent, which the authors indicate for some labial bracket-wire combinations and which deserves the characterization “irreal”, stresses the inappropriateness of the method of measurement.Further flaws include: a) incorrect interpretation of the measurement results, where a tooth tripping around (overcorrection) is interpreted as a better outcome than a perfect 100 per cent correction; b) using a statistical test in an inappropriate and misleading way; c) uncritical copying of text passages from older publications to describe the method, which do not correspond to this experimental protocol and lead to calculation errors; d) wrong citations; e)differences in table and bar graph values of the same variable; f) using a lingual mushroom shaped 0.013” Thermaloy-NiTi wire which does not exist; g) drawing uncritical conclusions of so called "clinical relevance" from a very limited in vitro testing.

**Conclusions:**

Clinical recommendations based on in vitro measurements using the Orthodontic Measurement and Simulation System (OMSS) should be read with caution.

## Background

### Bibliographic information and study summary

*Alobeid A, El-Bialy T, Reimann S, Keilig L, Cornelius D, Jäger A, Bourauel C. Comparison of the efficacy of tooth alignment among lingual and labial brackets: an in vitro study. Eur J Orthod. 2018;40:660-5.*

The in vitro study by Alobeid et al. was carried out using the so-called Orthodontic Measurement and Simulation System (OMSS) which was originally equipped with two sensors. The levelling and aligning stage of orthodontic treatment was simulated in vitro using a NiTi arch-wire (RMO, Denver, USA), ligated to brackets, which were bonded to an ideal occlusion model. In the area of the previously removed upper left first incisor, one sensor was fitted to the arch-wire. After a displacement of 2 mm horizontally or 2 mm vertically, a pathway back (correction) was simulated using a step-motor. In this in vitro simulation, the sensor was not moved backwards by the deflected arch-wire, but instead was moved backwards actively by the OMMS’s step-motor, in increments of 0.1 mm (OMSS driven, not arch-wire driven). The endpoint of the OMSS driven, backward movement was considered to have been achieved when no force generated by the arch-wire could be measured with the sensor. This endpoint was interpreted as being equal to the correction achieved by the arch-wire. The authors reported that the simulated malocclusion was corrected by all bracket systems (min. 12%, max. 138%). In the case of the maximum, a super-elastic, nickel-titanium arch-wire which had been deflected by 2 mm horizontally forward returned, on average, 2.7 mm horizontally backwards. In most measurements, on average, the thinner arch-wire achieved a larger correction value.

The authors concluded that lingual brackets were less efficient in initial tooth alignment than labial brackets. They also concluded that the thicker wire was not more efficient in initial tooth alignment than the thinner one. These purely clinical conclusions were drawn on the basis of in vitro measurements.

## Commentary and analysis

### Inadequate experimental design which leads to wrong measurements

**Fig. 1:**
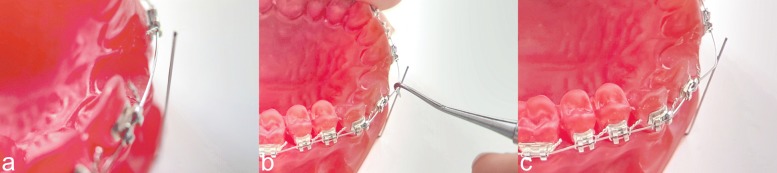
**a** An identical set up as in the study of Alobeid et al.: Acrylic resin model (Palavit G 4004; Heraeus Kulzer, Hanau, Germany) was fabricated from a duplicate of a Frasaco model (Frasaco, Tettnang, Germany) of a normal maxillary arch. The upper-right, central incisor was removed. The model was bonded with conventional brackets with 0.022” slot size (GAC Twin, Dentsply Sirona, Charlotte, USA). A 0.014” Thermaloy-NiTi arch-wire (RMO, Denver, USA) was inserted. The stainless steel ligatures used were tied using a needle holder. The ligature was first tightened around the bracket wings and then loosened by one turn, to allow free movement of the arch-wire. **b** The reference pin was placed at a distance of 2 mm from the arch-wire. **c** The simulation was carried out at an ambient temperature of 36^∘^C. A horizontal displacement of 2 mm was simulated. At the end of the displacement, the wire was stuck, because of friction and binding, and did not move back at all (correction = 0%). Alobeid et al. reported a correction of 1.6 mm, equal to 82%

**Fig. 2:**
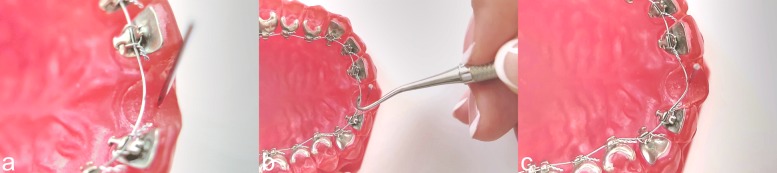
**a** An identical set up as in the study of Alobeid et al.: Acrylic resin model (Palavit G 4004; Heraeus Kulzer, Hanau, Germany) was fabricated from a duplicate of a Frasaco model (Frasaco, Tettnang, Germany) of a normal maxillary arch. The upper-right, central incisor was removed. The model was bonded with completely customized lingual brackets with a 0.018” slot size (Incognito, 3M Deutschland, Neuss, Germany). A 0.014” lingual NiTi arch-wire (RMO, Denver, USA) was inserted. As RMO only offers straight lingual arch-wires, these were used in the simulation. The stainless steel ligatures used were tied using a needle holder. The ligature was first tightened around the bracket wings and then loosened one turn, to allow free movement of the arch-wire. **b** The reference pin was placed at a distance of 2 mm from the arch-wire. **c** The simulation was carried out at an ambient temperature of 36^∘^C (sauna). A horizontal displacement of 2 mm was simulated. At the end of the displacement, the wire was stuck, because of friction and binding, and did not move back at all (correction = 0%). Alobeid et al. reported a correction of 0.6 mm, equal to 35%

The authors published quite astonishing measurement results: arch-wires "over-returning" to more than their state before elastic deformation; thinner wires achieving larger correction values than thicker wires. The most surprising outcome was, however, that these measurement results indicate that all wires tested returned, at least partly, to their pre-deformation shape. As is apparent from the videos (supplement: video 1 and 2) and Figs. 1 and 2, this was not the case in an identical experimental protocol for both the labial GAC TWIN bracket system (Dentsply Sirona, Charlotte, USA) and the lingual Incognito bracket system (3M Deutschland, Neuss, Germany). After a horizontal deflection by 2 mm, the arch-wires remained at their endpoints and did not return towards pre-activation at all. Also, during a post activation observation period of 120 minutes the wires did not move back. The same observations were made after a vertical deflection by 2 mm. Using an identical bracket-arch-wire combination, the authors Alobeid et al. measured a return of 1.6 mm (82%) for GAC TWIN and of 0.6 mm (35%) for Incognito after a horizontal deflection. The differences between the measured results and the in vitro reality (supplemented videos 1 and 2) can only be explained by assuming an inadequate experimental design (OMSS driven, not arch-wire driven) which did not allow the authors to represent correctly an evident physical process (the wire does not return to its original state because of friction and binding [1]). In addition to measurements which make little sense, the paper is riddled with errors and flawed information.

### Wrong citations in the introduction

**Fig. 3:**
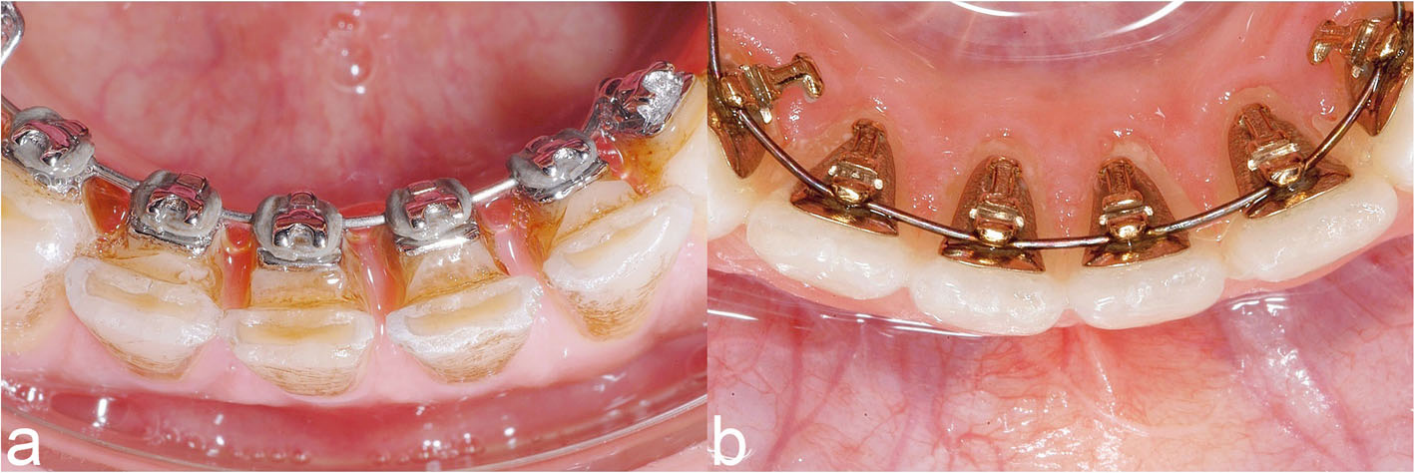
**a** Conventional lingual appliance. The conventional lingual brackets are individualized with a resin pad. The inter-bracket distance is very short. **b** Completely customized lingual appliance. The first arch-wire is routinely inserted behind the wings. The inter-bracket distance is substantially larger

In their introduction, the authors fail to give an adequate account of the current status of the lingual technique. This would appear to be so because the differences between conventional and completely customized lingual appliances have eluded them. In so doing, the content of sources referred to by them has been reported incorrectly: *"A fully customized lingual orthodontic appliance was introduced afterwards [...], and its results have been shown to be comparable to those of labial and regular lingual appliances (12)."* As to this, the authors wrongly reference a paper from 1986 [2], in which, however, no comparisons are made. Additionally, the referred literature with regard to the current biomechanical issues the lingual technique is confronted by, a book by Romano is referred to which is now over 20 years old [3]. The clinical relevance resulting from the introduction of completely customized lingual appliances have not come to the attention of any of the authors (Fig. 3). This is all the more surprising since these were expressly itemized in one of the papers the authors referenced themselves [4].

### Uncritical copying from older publications

In the materials and methods section, in particular, errors and flawed information appear. The authors do not even take the trouble to carefully describe how the measurements were made, but simply refer to earlier studies from the same source. Moreover, text passages and protocols sourced in earlier work by a similar group of authors are incorporated uncritically, which was bound to lead to quite obvious errors in taking measurements: *"In addition, a calculation of the tooth movement vector was mathematically analyzed considering the centre of resistance of the upper central incisor tooth to be located at 10 mm apically from the centre of the brackets and was located 4.5 mm palatally from the point of application of force..."* Analogously to the study by Montasser et al. [5], the centre of resistance of the relevant tooth, the upper left central incisor, is stated as being located 4.5 mm palatal from the bracket. The authors then go on and use this value in the computations. However, the uncritically reused text passage and protocol describes only the situation found in measurements for labial brackets. With lingual brackets, the resulting horizontal distance is substantially shorter (0 mm). For this reason, the subsequent measurements taken on lingual brackets not only make no sense, as indicated above, but are also flawed in their entirety.

### Testing arch-wires which do not exist

Furthermore, the lingual nickel-titanium arch-wires claimed to be used for the measurements are neither available from RMO (RMO, Denver, USA) in the stated size (0.013”) nor in the stated shape (mushroom): *"Two Thermalloy NiTi archwires 0.013-in and 0.014-in were used for all brackets. The transition temperature range (TTR) of thermalloy is 80−−90*^∘^*F (26.7−−32.2*^∘^*C). Regular archwires were applied for labial brackets and mushroom shaped lingual archwires were used with the lingual brackets (RMO, Denver, Colorado)."* In addition, the authors state: *"In our study, we have utilized 0.013” and 0.014” Thermalloy wires, which to our best knowledge are the commonly utilized initial levelling wires."* It is interesting to note that the manufacturers of the lingual appliances most frequently placed around the world do not even offer the 0.013” wire dimension.

### Incorrect interpretation of measurement results

The issue of overcorrecting is not addressed in some proper way by the statistical analysis as well as the discussion. Instead of reporting the absolute and relative correction (mm and %) it would have been appropriate to report the difference either absolute or relative to the ideal position (central incisor position). Neglecting this, the authors explain in the discussion that *"On the other hand, some passive SL brackets showed less correction than active labial SL brackets (e.g. the correction with FLI*^Ⓡ^* SL was 96 per cent compared to SPEED was 127 per cent in the z axis with 0.013” Thermalloy)."* Interpreted in this way, even massive overcorrections (tripping around) are always considered to be a better outcome.

The bar graph does not always depict what is presented in the tables (lingual bracket Joy™ [Adenta, Gilching, Germany]). Furthermore, the computation of the standard deviation percentages, too, is simply wrong in many instances, where this cannot be explained as the result of round-off errors.

### Misleading conclusions

With total disregard for the universally well-known high level of complexity associated with real-life tooth movements in the initial treatment stage, an in vitro study on a simulated malocclusion inexistent in reality (one tooth 2 mm outside or above the arch, no crowding, no spaces, ideal dental arch in all other respects) is turned into the basis of advocating clinical relevance. Furthermore, on that basis, a recommendation is made for one or the other system. Such a manner of proceeding, it would seem, can only be called dangerous and needs to be set right urgently, and not repeated in the future.

## Conclusion

Clinical recommendations based on in vitro measurements using the Orthodontic Measurement and Simulation System (OMSS) should be read with caution.

## Supplementary information

**Additional file 1** Video demonstrating that a return movement of a labial inserted 0.014” Thermaloy-NiTi arch-wire does not take place.

**Additional file 2** Video demonstrating that a return movement of a lingual inserted 0.014” NiTi arch-wire does not take place.

## Data Availability

Not applicable.
